# Maternal Social Support Reshapes the Offspring Brain Transcriptome to Buffer Prenatal Stress in Mice

**DOI:** 10.21203/rs.3.rs-10820227/v1

**Published:** 2026-09-07

**Authors:** Monserrat Rodríguez González, Paula Thomas, Lucía Janicki, Erika Georgieff, Sergio Nemirowski, Verónica I. Cantarelli, Marina F. Ponzio, Mariela Chertoff, Bruno G. Berardino, Eduardo T. Cánepa

**Affiliations:** 1 Laboratorio de Neuroepigenética y Adversidades Tempranas, Departamento de Química Biológica, Facultad de Ciencias Exactas y Naturales, Universidad de Buenos Aires, and Instituto de Química Biológica de Ciencias Exactas y Naturales (IQUIBICEN), CONICET, Ciudad Autónoma de Buenos Aires, Argentina.; 2 Departamento de Química Biológica, Facultad de Ciencias Exactas y Naturales, Universidad de Buenos Aires, and Instituto de Química Biológica de Ciencias Exactas y Naturales (IQUIBICEN), CONICET, Ciudad Autónoma de Buenos Aires, Argentina.; 3 Instituto de Fisiología Humana “Dr. Oscar Orías”, Facultad de Ciencias Médicas and Instituto de Investigaciones en Ciencias de la Salud (INICSA-CONICET), Universidad Nacional de Córdoba, Santa Rosa 1085, X5000ESU Córdoba, Argentina

**Keywords:** Affiliative and prosocial behaviors, Prenatal stress, Maternal care, Social support during pregnancy, Prefrontal cortex, Amygdala, Oxytocin system, Dopaminergic signaling, Long term spatial memory, maternal bedding preference

## Abstract

Early-life adversity, including prenatal stress, has lasting consequences for maternal and offspring mental health. However, the mechanisms through which social support buffers these effects remain poorly understood. Using a mouse model, we examined whether gestational social support from a familiar female protects against prenatal stress and whether this protection restores a stress-free molecular phenotype or engages a distinct compensatory program. Pregnant females exposed to unpredictable prenatal stress displayed impaired maternal care, elevated peripartum corticosterone levels, and reduced prefrontal and amygdala expression of oxytocinergic and dopaminergic genes. Their offspring showed impaired maternal-odor preference and long-term spatial memory. Strikingly, socially supported mothers and their offspring were virtually indistinguishable from unstressed controls across these behavioral and candidate-gene measures. Female companions increased affiliative interactions specifically in response to the pregnant female’s distress, consistent with a targeted prosocial response. However, whole-transcriptome analysis of offspring prefrontal cortex revealed a more complex picture: differentially expressed genes associated with stress and social support converged on synaptic transmission and neuronal connectivity, yet the transcriptome of socially supported offspring did not return to the control state. Instead, it diverged further along the same trajectory as prenatal stress, indicating an amplified rather than reversed molecular shift. These findings support a two-level model in which social support normalizes stress-reactive proximal signaling while activating a distinct adaptive transcriptional program in the developing brain. Maternal social support may therefore represent a modifiable factor that mitigates the intergenerational consequences of early-life adversity and reshape resilience as an active adaptation rather than restoration of baseline state.

## Introduction

Social species rely on prosocial behaviors to foster cooperation, strengthen social bonds, and improve group well-being. Among these, behaviors that provide comfort and mitigate stress in distressed individuals, such as gentle touch or vocal reassurance, are of particular significance^1,2^. alleviating negative emotional states in individuals who have experienced adversity^3,4^ and contributing to long-term resilience and emotional health in social groups^5^. In non-human animals, allogrooming plays a crucial role in mitigating stress: widely observed across mammals and birds, it increases specifically when a group member is distressed, providing a direct mechanism for stress alleviation^6,7^. Comparable behaviors reduce stress in humans by activating pathways involved in social bonding and emotional regulation^8,9^ These stress-buffering behaviors involve coordinated neural, endocrine, and behavioral systems. Key regions include the medial prefrontal cortex (mPFC), anterior cingulate cortex (ACC), and amygdala, involved in empathy and the recognition of distress in others^10-13^; the hypothalamic oxytocinergic system, which facilitates prosocial interactions and reduces stress responses^6,14^; and reward-related circuits, including the ventral tegmental area and nucleus accumbens, which reinforce comforting behaviors^15-17^.

This becomes particularly significant when the stressed individual is pregnant. Maternal stress during pregnancy is consistently linked, across large human cohort studies, to altered offspring neurodevelopment and increased risk of anxiety, depression, attentional problems, and other psychiatric conditions later in life, mediated by functional and structural brain changes, HPA-axis dysregulation, and epigenetic mechanisms^18-20^. Social support during pregnancy can buffer these effects on both mother and offspring: the maternal brain undergoes substantial plasticity during gestation, with regions such as the medial preoptic area of the hypothalamus becoming more sensitive to social input, and the developing fetus, highly responsive to maternal physiological states, appears to benefit from the stress-attenuating effects of the mother’s prosocial interactions, underscoring the intergenerational reach of social support^21,22^.

At the molecular level, prenatal stress produces lasting alterations in offspring gene expression and epigenetic regulation, modifying DNA methylation, non-coding RNA expression, and transcriptomic profiles in genes involved in neurodevelopment, synaptic function, and stress axis regulation^23-27^, thought to mediate the long-term behavioral and neurodevelopmental consequences of prenatal adversity^28,29^. However, whether protective interventions such as maternal social support act by reversing these molecular signatures toward an unstressed profile, or by engaging distinct compensatory mechanisms, remains largely unexplored — a question of particular interest in the mPFC and amygdala, given their established roles in stress regulation and the prosocial circuitry described above.

This study evaluates whether social support during early pregnancy protects against the effects of environmental adversity on maternal mental health and offspring behavioral outcomes of both sexes. We hypothesized that prosocial interactions during gestation would mitigate stress-induced impairments in maternal behavior and offspring neurodevelopment, rendering the socially supported group more similar to the unstressed condition, a prediction supported by our behavioral findings in dams and offspring. However, the underlying molecular mechanisms remain unclear, and at least two non-mutually exclusive models could account for this protection: social support may normalize the transcriptional alterations induced by prenatal stress toward the unstressed phenotype, or it may engage a distinct compensatory transcriptional program that diverges from both profiles yet still confers behavioral resilience.

Using a mouse model of prenatal stress, we integrated behavioral and molecular approaches to test whether supportive social interactions preserve maternal adaptation and promote resilient offspring neurodevelopment, and whether this protection reflects restoration of the basal molecular state or the establishment of an alternative adaptive transcriptional program in the offspring PFC.

## Methods

### Animals

CF-1 mice (crlfcen:CF1) from the colony of the Bioterio Central, Facultad de Ciencias Exactas y Naturales (University of Buenos Aires), were used for all experiments. Animals were maintained under controlled temperature (24 ± 1°C) and humidity (50 ± 10%), a 12:12 h light:dark cycle (lights on 6 a.m.), with food and water ad libitum,

### Experimental design

#### Formation of Dam–Support Female Pairs

Female mice were housed four per cage from 4 to 8 weeks of age, then divided into pairs of familiar, unrelated females housed independently (Fig 1). Affiliative and prosocial interactions between paired females were assessed as described below. After two weeks of pair housing (10 weeks of age), one female per pair was randomly selected for mating; the other, designated the support female, was housed individually with environmental enrichment. The day a vaginal plug was detected was designated gestational day 0 (GD0), and pregnant females were separated from males. From GD1 to GD12, pregnant females were exposed (ES, ESA) or not exposed (CTA) to unpredictable stressors, as detailed below. On GD13, the familiar support female was reintroduced into the home cage of ESA dams and remained until delivery; ES dams remained singly housed throughout.

#### Schedule of Prenatal Stressors

Prenatal stress was applied daily from GD1 to GD12. Four validated stress procedures were used, adapted from established rodent models^30,31^: swimming (5 min in room-temperature water), restraint (20 min in a ventilated Plexiglas restrainer), shaking (5 min of low-speed vortex shaking), and reduced bedding, applied continuously throughout. One of the three intermittent procedures was applied daily in an unpredictable, alternating sequence between 08:00 and 16:00 h in a room separate from housing (full procedural parameters in Supplementary Material).

#### Ethical Considerations

A control group without social support (CT) was not included in this design, as prior experiments from our laboratory found no significant differences between CT and CTA animals across multiple developmental and behavioral endpoints; CTA was therefore used as the control condition throughout, consistent with the 3Rs principle of reduction and the primary aim of testing whether social support buffers the effects of prenatal stress (extended justification in Supplementary Material).

### Assessment of Affiliative and Prosocial Interactions Between Dams and Support Females

Affiliative and prosocial behaviors were assessed in pairs of a future dam and support female (unrelated) across two phases: basal interaction, evaluated for 8 consecutive days prior to mating, and post-stress interaction, reassessed from GD13 to delivery following reunification of the pair. In both phases, behavior was scored twice daily (08:00, 16:00 h; 1 h per session) using instantaneous sampling every 3 min, classified as active affiliative behaviors (allogrooming, following, and sniffing), passive interactions (physical contact without overt behavioral activity), or non-contact events. Sessions were video-recorded, and behavioral scoring was performed offline by an observer blind to group assignment (CTA or ESA).

### Maternal care

Maternal care was evaluated from PD1 to PD6 as previously described^32^, with dams observed for three 1-h periods daily (9 am, 1 pm, 4 pm) and activity scored sequentially every 3 min across categories including arched-back nursing (ABN), licking/grooming (LG) of pups, passive nursing, carrying pups, mother/pup contact, nest building, and other maternal and non-maternal behaviors (full ethogram in Supplementary Material). Scoring for every cage was performed by three observers trained to a high level of inter-rater reliability.

### Corticosterone Determinations in Feces

Corticosterone determination in feces was performed as described previously^33^. Detailed procedures are provided in the Supplementary Material

### Behavioral testing

Dams and support females underwent a standardized behavioral battery comprising the elevated plus maze^34^ (EPM), novel object recognition^35^ (NOR), three-chamber social interaction test^36^ (SI), and forced swim test^37^ (FST), with 4-day intervals between tests. Detailed procedures are provided in the Supplementary Material.

### Maternal Bedding Preference Test

Maternal odor-based preference was assessed in offspring at PD14-PD15 by exposing pups to bedding from their home cage and from an unfamiliar dam’s cage (full apparatus and procedure in Supplementary Material). Preference was quantified as a discrimination index: (T_maternal - T_non-maternal) / (T_maternal + T_non-maternal), where T reflects time spent exploring each bedding source.

### Four-hole board test

Long-term spatial memory was assessed in offspring of both sexes using a four-hole board test over two consecutive days^38^ (full apparatus and procedure in Supplementary Material). Nose pokes during session 1 indexed exploratory behavior, and the decrease in nose pokes at session 2 (24 h later) indexed long-term spatial memory.

### RNA isolation and reverse transcription

Mice were killed by cervical dislocation; the mPFC and amygdala were rapidly collected, snap frozen in liquid nitrogen, and stored at −80°C. Total RNA was extracted from frozen tissue using RNAzol RT (MRC, OH, USA) according to the manufacturer’s instructions. RNA concentration and purity were assessed using a NanoDrop2000 (Thermo Fisher Scientific, MA, USA), and cDNA was synthesized as previously described^39^.

### Gene expression analysis

Primers (Primer-BLAST, NIH; Table 1) were used for RT-qPCR (StepOnePlus, Thermo Fisher Scientific) in duplicate, with non-template and non-transcribed controls included on each plate^40^. Differential expression was assessed from initial template quantities (N0) estimated by linear regression (LinRegPCR), normalized to the geometric mean of Hprt expression^41^.

### RNA sequencing and differential expression analysis

Paired-end, demultiplexed, adapter-trimmed FASTQ files were obtained from Novogene (USA); quality and contaminants were assessed with FastQC/MultiQC and FastQ Screen^42,43^. Reads were aligned to GRCm39 with HISAT2^44^, and gene-level counts generated with featureCounts (Rsubread; Ensembl v112)^45,46^, filtering low-count genes with edgeR’s filterByExpr^47,48^. Differential expression was assessed with DESeq2; sex-by-group interactions were evaluated by likelihood ratio test, and ashr shrinkage was applied to improve log2 fold-change estimates^49^. To assess the role of differentially expressed lncRNAs, we applied a co-expression-based guilt-by-association strategy^50,51^. Male and female offspring were analyzed jointly, with sex included as a covariate (sequencing quality metrics in Supplementary Material). Overrepresentation analysis (ToppGene Suite/ToppFun) was performed against a custom background of expressed protein-coding genes (baseMean > 10) for GO Biological Process, Molecular Function, and Cellular Component, with redundant terms summarized using REVIGO. Pre-ranked GSEA was performed with fgsea in R^52^ using GO Biological Process gene sets from MSigDB (v2026.1, mouse; enrichment parameters in Supplementary Material).

### Statistical analysis

Statistical analyses were performed using GraphPad Prism v9 and R. Data were tested for normality using the Shapiro-Wilk test; normally distributed data were analyzed using parametric tests, and non-normally distributed data using non-parametric tests. For comparisons between two independent groups, unpaired two-tailed Student’s t-tests or Mann-Whitney U tests were used as appropriate. For comparisons among the three experimental groups, one-way ANOVA or Kruskal-Wallis tests were performed according to data distribution, followed by Tukey’s multiple-comparisons test or Dunn’s post-hoc test, respectively. For analyses involving sex and treatment, two-way ANOVA assessed the main effects and their interaction. For longitudinal measurements, repeated-measures mixed-effects models were used, with treatment, time, and their interaction as fixed effects, followed by Tukey’s post-hoc comparisons where appropriate. The same approach was used for maternal care measures collected across postpartum days. For categorical variables, chi-square tests were used, with effect sizes reported as Cramer’s V and odds ratios with 95% confidence intervals; one-sample t-tests were used when performance was compared with a theoretical or within-animal reference value. Effect sizes were calculated where relevant and reported as η^2^, ω^2^, Cohen’s d, ε^2^, Cramer’s V, or odds ratios, depending on the test and outcome measure. Tests were two-sided, with significance defined as p < 0.05 and trends indicated between p > 0.05 and p < 0.1 (full test-by-outcome assignment in Supplementary Material). For RNA-seq, differential expression was assessed using a negative-binomial generalized linear model (DESeq2), with differentially expressed transcripts defined at an adjusted p-value (Benjamini-Hochberg FDR) ≤ 0.10; nominally significant genes (p < 0.05) were additionally examined for the ES-versus-CTA and ESA-versus-CTA comparisons to characterize broader transcriptional shifts. GSEA pathways were considered enriched when ∣NES∣ > 2 and FDR < 0.05.

## Results

### Prenatal stress induces increased prosocial interactions within female dyads

To determine whether prenatal stress alters social interactions between pregnant females and their support partners, we quantified active, passive, and non-interaction events in CTA and ESA dyads at baseline (before pregnancy and stress) and during pregnancy following stress exposure.

At baseline, no differences were detected between CTA and ESA pairs in active (U = 37.50, p = 0.6129), passive (t(17) = 1.402, p = 0.1789), or non-interaction events (t(17) = 1.080, p = 0.2954) (Figs. 2A-C), confirming the absence of assignment bias. During pregnancy and following stress exposure, ESA females showed a significant increase in both active (t(17) = 2.314, p = 0.0334) and passive interactions (t(17) = 3.588, p = 0.0023) with their partners relative to CTA females, and fewer non-interaction events (t(17) = 2.401, p = 0.0281) (Figs. 2D-F). Because this increase emerged specifically after stress exposure, it likely reflects a prosocial response directed toward a distressed pregnant female rather than an effect of pregnancy per se, which was shared by both groups.

### Social support attenuates prenatal stress-induced deficits in active maternal care

We next evaluated whether prenatal stress affected maternal care and whether social support modified these behaviors. Overall pup-directed activity did not differ among groups (F(2,24) = 1.242, p = 0.3068) (Fig. 3A). However, specific components differed: ES dams showed reduced ABN (H(2) = 11.77, p = 0.0028) and LG (H(2) = 8.331, p = 0.0155), whereas ESA dams showed levels comparable to CTA (Figs. 3B and C). Analysis of ABN+LG across the observation period revealed a main effect of treatment (F(2,24) = 7.016, p = 0.0040) with no treatment x time interaction (F(10,120) = 1.496, p = 0.1488); ABN+LG was reduced in ES dams on postnatal days 2 and 5 (Tukey’s p = 0.0071 and p = 0.0446) (Fig. 3D).

No differences were observed in passive nursing (H(2) = 2.495, p = 0.2872), nest building (H(2) = 1.949, p = 0.3773), or contact with pups in the nest (F(2,24) = 1.236, p = 0.3083) (Fig. S3A-C). In contrast, behaviors not involving the offspring differed among groups (F(2,24) = 3.490, p = 0.0467), with ES dams tending to spend more time in these activities than CTA and ESA dams (Tukey’s p = 0.0864 and p = 0.0694) (Fig. 3E); self-maintenance activities did not differ (F(2,24) = 1.857, p = 0.1779) (Fig. S3D).

Together, these findings indicate that prenatal stress reduced the quality, rather than the amount, of maternal care, whereas ESA dams displayed care comparable to CTA despite the same stress exposure, consistent with a protective effect of social support.

Additional reproductive, phenotypic, and behavioral assessments (maternal and offspring body weight, litter size, offspring survival, and maternal and social-support performance in the EPM, FST, NOR, and three-chamber test) are provided in the Supplementary Material.

### Fecal corticosterone levels across the reproductive and postpartum period

Fecal corticosterone was monitored longitudinally in a subset of animals per group at five time points spanning key reproductive and postpartum transitions. Concentrations did not differ among groups before mating (H(2) = 0.93, p = 0.6682) or after the 12-day stress exposure (H(2) = 0.46, p = 0.8247) (Figs. S7A and B). On the day of delivery, a significant group effect emerged (H(2) = 7.00, p = 0.0062, epsilon squared = 0.55), with ES animals showing higher corticosterone than CTA (p = 0.0349) while ESA did not differ from CTA (p = 0.2147) (Fig. S7C). At PD7, a group effect was again detected (H(2) = 6.64, p = 0.0199, epsilon squared = 0.52), with trends toward higher corticosterone in ES relative to CTA (p = 0.089) and ESA (p = 0.069) that did not survive Holm correction (Fig. S7D). By weaning, groups no longer differed (H(2) = 1.10, p = 0.6149) (Fig. S7E). Support females showed no CTA-versus-ESA differences at either the pre-stress (U = 5, p = 0.2857) or post-stress time point (U = 4, p = 0.3429) (Figs. S7F and G).

### Prenatal Stress-Induced Changes in Prosociality-Related Gene Expression in the PFC and Amygdala Are Modulated by Social Support

Given the increased affiliative interactions in ESA dyads, we examined whether expression of genes implicated in social buffering and socio-emotional regulation (oxytocinergic, dopaminergic, serotonergic, and activity-dependent signaling) was altered in the PFC and amygdala.

In ES mothers, *Oxtr* and *Cd38* were reduced in the PFC relative to both CTA and ESA (Figs. 4A and B), and *Oxtr* was reduced in the amygdala relative to CTA, with a trend relative to ESA (Fig. 4G). *Comt* was reduced in ES mothers in both regions relative to CTA and ESA (Figs. 4D and J), and *Drd2* in the PFC only (Figs. 4C and I); no CTA-versus-ESA differences were detected for any of these genes. *Slc6a4* did not differ among groups in either region (Figs. 4E and K). For *Npas4*, an activity-dependent immediate early transcription factor, no differences were detected in the PFC (Fig. 4F), whereas a trend toward reduced expression was observed in the ES amygdala relative to CTA (Fig. 4L).

In social-support females, no group differences were detected for any gene in either region (Figs. S8A-L), except a trend toward reduced *Slc6a4* in the amygdala of ESA relative to CTA support females (Fig. S8K).

Overall, prenatal stress was associated with reduced expression of multiple genes implicated in affiliative and prosocial behaviors in the PFC and amygdala, whereas ESA mothers showed no detectable alterations relative to CTA, consistent with a modulatory effect of social support on stress-associated molecular changes in socio-emotional circuits.

### Prenatal stress disrupts maternal bedding preference in offspring and is prevented by social support

We next investigated whether the reduced maternal care in ES dams was associated with offspring behavioral alterations. Male and female pups (PD14-PD15) were exposed for 6 min to bedding from their own and an unfamiliar dam to assess maternal odor preference. A two-way ANOVA revealed no sex x treatment interaction (F(2,42) = 0.2975, p = 0.7442) and no main effect of sex (F(1,42) = 0.0488, p = 0.8261) (Fig. S9A), so sexes were pooled. The discrimination index differed among groups (H(2) = 6.024, p = 0.0492) (Fig. 5A), being lower in ES than CTA offspring (Dunn’s p = 0.0168) while ESA did not differ from CTA (p = 0.1390); effect sizes were moderate (eta squared = 0.092; CTA-versus-ES Cohen’s d = 0.66; ES-versus-ESA d = 0.56). One-sample t-tests confirmed that CTA (p = 0.0006) and ESA (p = 0.0043) offspring preferred their own maternal bedding, whereas ES offspring showed no preference (p = 0.3453); total exploration time did not differ among groups (H(2) = 2.727, p = 0.2557), indicating the effect was not attributable to overall exploratory activity (Fig. 5B). Thus, prenatal stress disrupted maternal bedding preference, and social support prevented it.

### Long-Term Spatial Memory and Exploratory Strategy in the 4-Hole Task

Offspring were tested in the 4-hole task, with retention assessed as the change in nose-pokes from training (day 1) to testing (day 2). A two-way ANOVA revealed a main effect of treatment (p < 0.0001), with no effect of sex (F(1,52) = 1.711, p = 0.1966) or sex x treatment interaction (F(2,52) = 0.7738, p = 0.4665) (Fig. S9B), so sexes were pooled. The day 2 versus day 1 difference in nose-pokes differed among groups (F(2,55) = 10.64, p < 0.001) (Fig. 5C), with differences between CTA and ES (Tukey’s p = 0.002) and between ESA and ES (p = 0.0039) but not CTA and ESA (p = 0.6496). CTA and ESA offspring significantly reduced their nose-pokes on day 2 (p = 0.0002 and p = 0.027), whereas ES offspring did not (p = 0.9370), indicating impaired memory retention in the ES group.

Exploratory motivation during training was assessed from holes visited on day 1, classifying animals as high (three-four) or low explorers (one-two). The distribution showed a trend toward a treatment effect (chi square(2, N = 69) = 4.85, p = 0.088, Cramer’s V = 0.27) (Fig. 5D): CTA offspring were predominantly high explorers (19/24) and ESA resembled them (17/22), whereas ES offspring showed more low explorers (11/23). Pairwise analysis showed a trend toward fewer high explorers in ES than CTA (OR = 3.48, 95% CI 0.97-12.54, p = 0.069), with no CTA-versus-ESA (OR = 1.12, p = 1.000) or ES-versus-ESA difference (OR = 0.32, p = 0.120). Thus, prenatal stress impaired long-term spatial memory and was associated with a trend toward reduced exploratory engagement, whereas social support prevented the memory impairment and yielded an exploratory profile resembling controls.

### Distinct transcriptomic signatures associated with prenatal stress and social support in the offspring prefrontal cortex

To investigate molecular mechanisms underlying the offspring behavioral phenotype, we performed whole-transcriptome RNA sequencing of the PFC (sexes analyzed jointly, with sex as a covariate). The ES-versus-ESA comparison identified 625 differentially expressed transcripts (padj <= 0.1), including 343 protein-coding genes, 76 long non-coding RNAs (lncRNAs), and a smaller number of transcripts from other biotypes. Overrepresentation analysis of the 343 protein-coding DEGs against a custom background of expressed protein-coding genes (baseMean > 10) was performed for GO Biological Process (BP), Molecular Function (MF), and Cellular Component (CC); of the 343 query genes, 325 were recognized. All three domains converged on synaptic transmission and neuronal connectivity: BP was led by chemical synaptic transmission (52/325 genes; FDR = 8.62x10-7), together with dendritic spine organization and development and behavior (Fig. 6A); MF highlighted neurotransmitter receptor and channel activity, particularly GABAergic signaling (Fig. 6B); and CC showed the strongest support, led by glutamatergic synapse (54/325 genes; FDR = 2.68x10-8), followed by GABAergic synapse, presynapse, postsynaptic specialization, and axonal compartments (Fig. 6C).

Among the 76 differentially expressed lncRNAs, a co-expression-based guilt-by-association approach (Guttman et al., 2009; Liao et al., 2011) identified 975 lncRNA-gene pairs involving 619 unique protein-coding genes and 36 of the 76 lncRNAs. Kcnq1ot1 showed robust correlations with three protein-coding genes, Dpysl2, Lars2, and Xkr6; Dpysl2, which encodes CRMP2, has established roles in axon guidance and synaptic plasticity, consistent with the functional enrichment of the protein-coding DEG set.

#### Comparison across experimental groups

To characterize transcriptional differences across groups, we examined the ES-versus-CTA and ESA-versus-CTA contrasts. ES-versus-CTA yielded 417 nominally significant genes (p < 0.05) but none reaching adjusted significance, whereas ESA-versus-CTA yielded 1,507 nominally significant genes, of which Tspyl5 and Epm2a reached adjusted significance (both padj = 0.038). Among the 73 genes showing nominal differences in both contrasts, 93.2% changed in the same direction (r = 0.87 for shrunken log2 fold-change estimates), and the magnitude of change was greater in ESA-versus-CTA than ES-versus-CTA for all 73 genes. Thus, the ESA transcriptome did not simply return to the CTA state but showed a broader shift along the same direction as the ES-versus-CTA contrast.

GSEA of the three pairwise comparisons (∣NES∣ > 2, FDR < 0.05) revealed that almost all enriched pathways were detected in a single direction within each comparison: ES-versus-CTA yielded 59 pathways with NES < −2 and none with NES > 2; ES-versus-ESA yielded 112 with NES < −2 and two with NES > 2; and ESA-versus-CTA yielded 120 with NES > 2 and two with NES < −2. Classification of the top 50 enriched pathways revealed distinct functional profiles: ES-versus-CTA was characterized mainly by lipid metabolism and membrane biosynthesis, together with glial development and nervous tissue organization (Fig. S10A); ES-versus-ESA was dominated by synaptic organization and function, with additional categories related to intracellular trafficking, proteostasis, neuronal development, and structural plasticity (Fig. S10B); and ESA-versus-CTA showed a more homogeneous profile dominated by dendritic spine development, structural plasticity, and synaptic organization (Fig. S10C).

These findings support the hypothesis that prenatal social buffering promotes behavioral recovery through the establishment of an alternative transcriptomic program rather than by restoring the transcriptome to the basal control state.

## Discussion

This study explored whether social support during gestation buffers the consequences of prenatal stress in dams and their offspring, and whether this protection operates by restoring the unstressed molecular phenotype or by a distinct compensatory mechanism. Our findings support a nuanced answer: depending on the level of biological organization examined, both models find support, and reconciling them offers a more complete picture of how social buffering operates across timescales and biological systems.

The unpredictable stress model used here did not induce overt anxiety-like or depression-like phenotypes, nor did it impair recognition memory or sociability in adult dams. Instead, its effects were remarkably selective, primarily affecting maternal caregiving quality and neural systems associated with affiliative behavior, together with developmental outcomes in the offspring, consistent with evidence that moderate gestational stress primarily disrupts maternal-offspring interactions and offspring brain development rather than inducing severe affective disturbances in the mother^18,53,54^. This selectivity was also evident in the companion females’ own behavior: the rise in prosocial interactions was not a general effect of cohousing or pregnancy, but was specifically linked to the companion’s exposure to stress, a pattern that aligns with the literature on rodent consolation and allogrooming, in which affiliative behaviors are heightened by a partner’s distress rather than by mere proximity^6,55^.

The distinction between quantitative and qualitative aspects of maternal care is biologically important, as caregiving components differ in their neurobiological significance^56^. Arched-back nursing facilitates pup access to milk while maintaining thermal and tactile contact, and its disruption is linked to poorer nursing efficiency and altered offspring outcomes, whereas licking and grooming provide intense somatosensory input that shapes sensory pathway maturation, HPA-axis regulation, and synaptic development^57-60^. Reductions in these behaviors are therefore thought to exert disproportionately large developmental effects despite relatively preserved overall maternal contact.

Our finding that ES dams show reduced maternal care alongside elevated peripartum corticosterone, both buffered in ESA, and that ES offspring in turn show impaired maternal-odor preference and long-term spatial memory, again buffered in ESA, suggests that maternal behavior may be a proximate transmission route for the protective effect of social support. This is consistent with the classical concept of social buffering, whereby an affiliative conspecific reduces HPA-axis activation during or following stress^61,62^, a phenomenon documented across rodents, primates, and humans and thought to involve oxytocinergic pathways, limbic circuits, and hypothalamic stress-regulatory networks^63-65^. The temporal profile was particularly informative: group differences in corticosterone were evident during late gestation and the first postpartum week but disappeared by weaning, indicating that social support acted within a sensitive window rather than chronically suppressing HPA-axis function. This specificity may be advantageous, as excessive glucocorticoid exposure during late gestation has been repeatedly implicated in fetal programming through altered placental function, fetal brain development, and maturation of stress-regulatory circuits^66,67^.

The molecular data are consistent with disruption of oxytocinergic and dopaminergic cortical-limbic systems involved in maternal responsiveness^68,69^. Oxytocin signaling contributes to the onset and maintenance of maternal care and interacts with mesolimbic dopamine pathways supporting maternal motivation and social reward, while dopamine reinforces pup-directed actions^57,70,71^. The preservation of both maternal behavior and candidate-gene expression in socially supported dams is therefore consistent with social support stabilizing stress-sensitive circuits during pregnancy.

Notably, these alterations occurred in two regions central to social behavior, the mPFC and amygdala, whose functional interactions are increasingly recognized as fundamental to social buffering, allowing affiliative cues to influence endocrine and behavioral stress responses^72,73^. Their conservation provides a plausible neural substrate through which social interactions maintain maternal adaptation during pregnancy^74,75^.

The protective effects extended beyond the mother to the next generation. The conservation of maternal bedding preference is particularly noteworthy, as attraction to maternal olfactory cues is one of the earliest manifestations of successful mother-infant bonding in rodents: maternal odors guide nipple localization, nursing, thermoregulation, and proximity-seeking, promoting survival while shaping the maturation of circuits involved in attachment and emotional regulation^76,77^. Consequently, the absence of maternal odor preference in offspring from stressed dams likely reflects disruption of early socio-sensory processing rather than a simple alteration in olfactory perception.

The impairment in long-term spatial memory in offspring from stressed dams is consistent with evidence that prenatal adversity alters the maturation of corticolimbic circuits involved in learning and memory^78,79^. These deficits were prevented by maternal social support, indicating that the prenatal social environment can influence cognitive trajectories long after birth^80,81^. A further, non-significant observation was a tendency toward reduced exploratory motivation in prenatally stressed offspring; because exploratory motivation influences opportunities for environmental interaction and learning, even subtle changes may contribute to broader cognitive outcomes later in life^82,83^. These alterations emerged despite the absence of detectable sex differences: although many studies report sex-dependent effects of prenatal stress34,35, increasing evidence indicates that some consequences, particularly those involving sensory processing, hippocampal-dependent memory, and prefrontal maturation, can be similarly expressed in both sexes depending on the nature, timing, and intensity of the prenatal insult^84,85^.

A markedly different picture emerged from the unbiased, whole-transcriptome analysis of the offspring prefrontal cortex, which revealed broad molecular differences associated with prenatal stress and buffering, consistent with prior evidence that prenatal stress induces lasting transcriptomic and epigenetic reprogramming in fetal and adult brain^86,87^. The interpretation that buffered offspring do not simply revert to control baseline but instead occupy an alternative molecular state is plausible given evidence that stress-related phenotypes can arise from coordinated, pathway-level shifts, including lncRNA-linked regulation, rather than only large single-gene effects^88^. The impaired long-term memory in ES offspring, and its recovery in ESA offspring, is consistent with the differential expression of genes governing dendritic spine organization and development and glutamatergic synaptic transmission identified here^79,89^. The acquisition of preference for maternal bedding, an early form of odor-guided social learning, similarly depends on the maturation of synaptic connectivity within limbic and sensory-processing circuits during a comparable developmental window; the reduction in genes supporting synaptic transmission and spine organization thus offers a plausible, if indirect, molecular substrate for the disrupted preference in ES offspring^90^.

Reconciling these two patterns, restoration at the level of candidate social-affiliation genes and maternal/offspring behavior versus divergence at the level of the broader offspring transcriptome, suggests a plausible two-tier model of social buffering. Social support during gestation may first normalize proximal signaling systems directly tied to social bonding and stress reactivity (the oxytocinergic and dopaminergic pathways examined here), both in the dam and, downstream, in her offspring. This normalization may in turn permit, or actively engage, a distinct and more pronounced program of synaptic and neuronal gene expression in the offspring brain, one that diverges from both stressed and unstressed profiles yet is associated with preserved behavioral outcomes. Under this model, behavioral resilience would not stem from erasing the molecular signature of stress, but from the recruitment of an additional, adaptive layer of neurodevelopmental regulation superimposed on a partially normalized signaling substrate, consistent with human evidence that social support during pregnancy buffers the impact of prenatal stress on offspring outcomes even when the underlying pathways remain unresolved^91^.

These findings carry broader translational implications. In humans, perceived social support during pregnancy consistently predicts higher-quality mother-infant interactions, greater maternal sensitivity, and improved cognitive and emotional development in children, even after accounting for socioeconomic and obstetric variables^92-94^, and structured support delivered through community doulas or peer programs reduces postpartum depression, anxiety, and stress in randomized controlled trials, particularly among socially vulnerable populations^95,96^. Although extrapolation from rodents to humans requires caution, this cross-species convergence supports the notion that social relationships during pregnancy shape offspring development both by buffering maternal stress physiology and by preserving the quality of early caregiving. The present data provide mechanistic support by linking social support to endocrine regulation, maternal brain molecular signatures, caregiving behavior, and offspring neurobehavioral outcomes within a single integrative model.

Equally informative is the absence of detectable molecular changes in the brains of the social-support females. Despite participating in increased affiliative interactions with stressed dams, support females showed no consistent alterations in candidate gene expression, suggesting that the neurobiological consequences of social buffering are not necessarily shared equally between interacting individuals: the primary effect appears to occur within the stressed animal, while the supporting conspecific may function as a relatively stable source of social regulation. This asymmetry resembles observations in human social neuroscience, where supportive individuals often facilitate emotional regulation in distressed partners without exhibiting parallel physiological responses themselves^97-99^.

### Strengths and limitations

A major strength of this study is its integrative design, combining maternal endocrine and behavioral measures with offspring behavioral and prefrontal transcriptomic analyses across two generations, providing convergent evidence for the biological consequences of prenatal social support; the use of a naturalistic affiliative paradigm also increases translational relevance. Several limitations should nevertheless be considered. First, transcriptomic analyses were restricted to the prefrontal cortex and a single developmental time point, so the identified changes may represent only part of the broader neural adaptations induced by the prenatal social environment. Second, the design does not establish causal relationships among maternal physiology, behavior, and offspring outcomes, or distinguish prenatal effects from postnatal influences mediated by maternal care; cross-fostering or targeted manipulation of maternal social interactions would be required to disentangle these mechanisms. Third, RNA sequencing identifies transcriptional associations but does not establish the cellular origin or functional consequences of the observed changes, which will require cell-type-specific and spatial approaches together with functional validation. Finally, although both sexes were included, the study was not powered to detect subtle sex-dependent molecular effects, and larger sex-stratified studies will be important to determine whether the mechanisms underlying resilience differ between males and females.

### Conclusion

This study provides converging behavioral, physiological, and molecular evidence that social support during gestation buffers the maternal and offspring consequences of prenatal stress. Our findings suggest that social buffering operates differently in the mother and the developing offspring brain. In mothers, social support exerted a predominantly homeostatic effect, preserving endocrine regulation, candidate gene expression, and maternal behavior in the face of prenatal stress. In contrast, although offspring behavioral outcomes were largely normalized, prefrontal transcriptomic profiles were not simply restored to the basal state. This dissociation suggests that, in the developing brain, resilience may not depend on preventing molecular adaptation, but rather on redirecting developmental trajectories toward an alternative adaptive state compatible with preserved behavioral function. These findings underscore social support as a modifiable, translationally relevant target for interventions aimed at mitigating the intergenerational consequences of early-life adversity.

## Supplementary Material

This is a list of supplementary files associated with this preprint. Click to download.

• Supplementalmaterial.docx

## Figures and Tables

**Figure 1.**
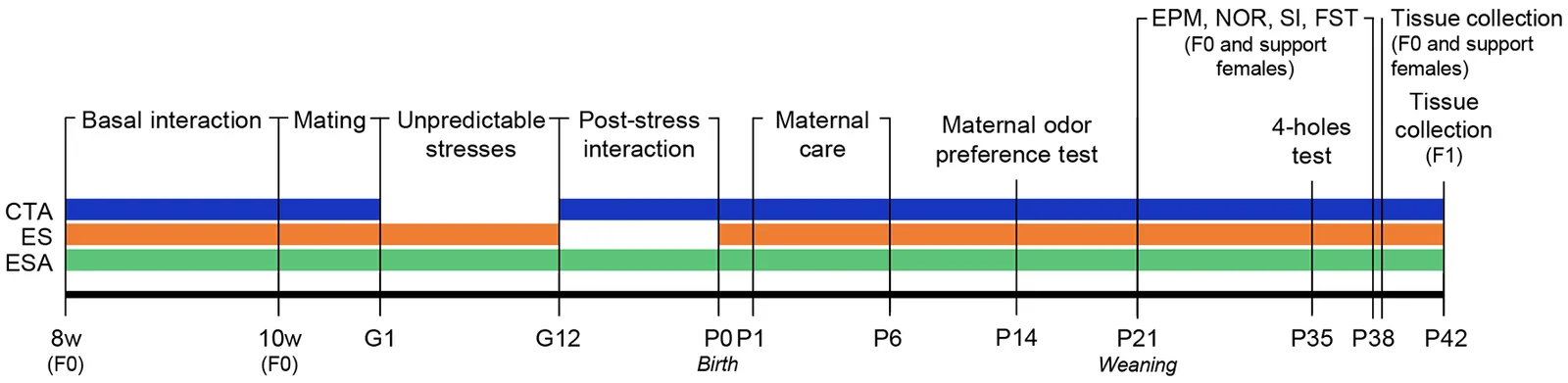
Experimental design of the social support model. Schematic representation of the experimental timeline used. Female mice (F0) were assigned to three experimental groups: control (CTA), prenatal stress (ES), or prenatal stress with social support (ESA). Before mating, basal social interactions between pregnant females and their designated support females were assessed. Following mating, females in the ES and ESA groups were exposed to unpredictable stressors during pregnancy. After the stress period, social interactions between ESA dams and their designated support females were assessed during the post-stress period. Maternal care was evaluated during lactation. Offspring (F1) were assessed for maternal odor preference at P14 and spatial memory using the 4-hole test at P35. Between weaning and P38, F0 dams and support females were evaluated using the Elevated Plus Maze (EPM), Novel Object Recognition (NOR), Social Interaction (SI), and Forced Swim Test (FST). Tissue collection was performed at the indicated time points from F0 dams and support females, as well as from F1 offspring. Birth and weaning are indicated at P0 and P21, respectively. G1 and G12 indicate the beginning and end of the exposure to unpredictable stress, respectively. PD: postnatal day; GD: gestational day.

**Figure 2.**
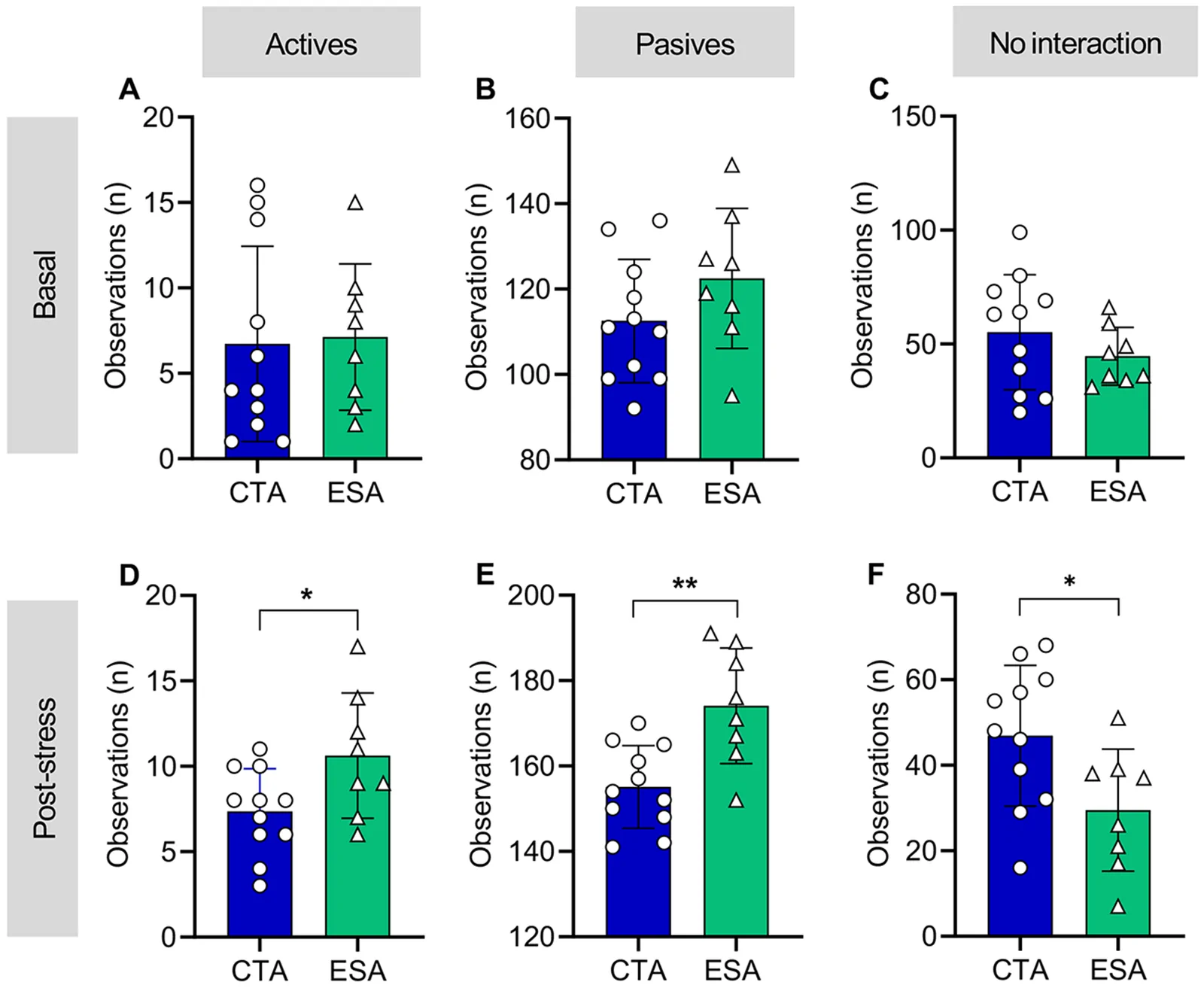
Social interaction. Social interactions between pregnant females and their designated support partner were assessed twice daily during two experimental phases: baseline, prior to mating (**A–C**), and post-stress, between gestational days PD13 and PD20 (**D–F**). Active interactions are shown in panels **A** and **D**, passive interactions in panels **B** and **E**, and absence of interaction in panels **C** and **F**. The total number of observations is indicated in each panel. Data are presented for the CTA group (n = 11) and the ESA group (n = 8). Statistical analysis was performed using an unpaired Student’s t-test. *p < 0.05, **p < 0.01.

**Fig. 3:**
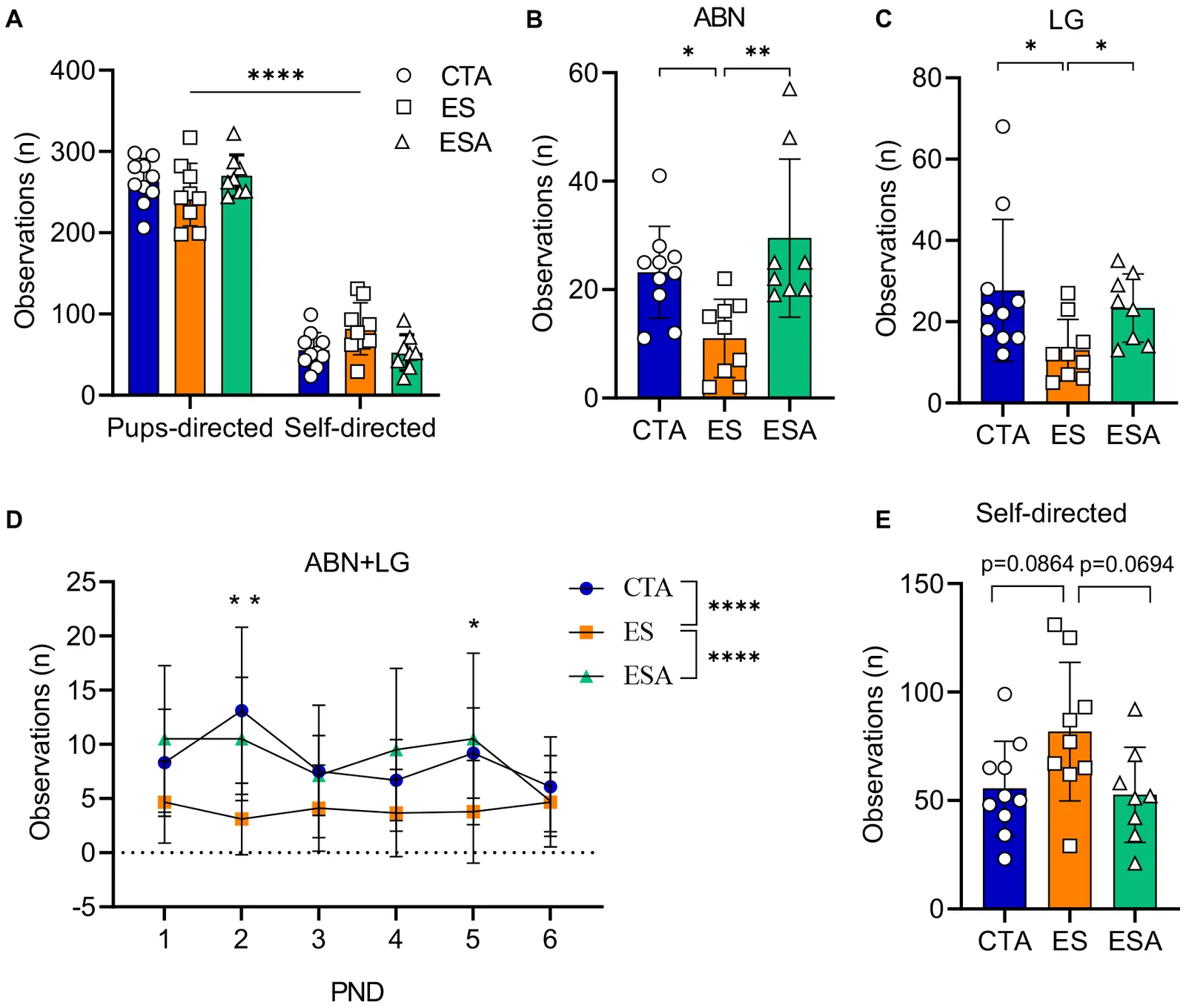
Analysis of maternal behavior. Maternal behavior was assessed three times per day every other day from PND1 to PND6. The following parameters were measured: maternal behaviors directed to the pups or self-directed (**A**), ABN (**B**), LG (**C**), and self-directed activities (**E**). The total number of observations are shown in each panel. ABN plus LG are shown across the observation period (**D**). The data are presented as the mean ± SD of an average of observations from all days (panels **A, B, C**, and **E**) or from each day (panel **D**), n = 8-9 per group. Statistical analysis for panel (**A**): Effect of behavior direction p < 0.0001 (two-way ANOVA), independently of gestational exposure. Statistical analysis for panels (**B, C,** and **E**): *p < 0.05 , **p < 0.01, one-way ANOVA. Statistical analysis for panel (**D**): *p < 0.05, **p < 0.01 two way ANOVA with repeated measures. Statistical trends are indicated by adjusted p values

**Figure 4.**
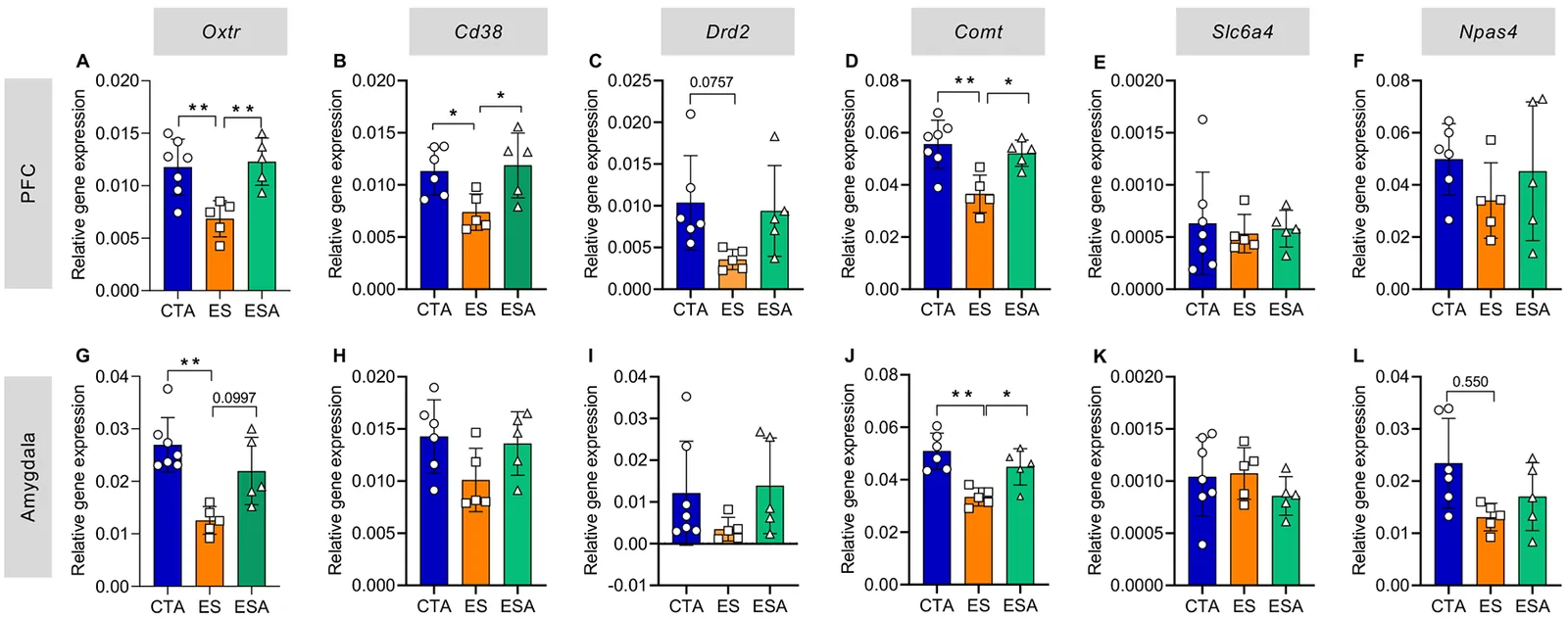
Expression of prosociality-related genes in the PFC and amygdala of mothers. Relative expression levels of the genes indicated in the figure were determined by RT-qPCR in the PFC and amygdala of mothers. Data are presented as mean ± SD (n = 5–7 per group). Statistical analysis for panels **E** and **G** was performed using the Kruskal–Wallis test followed by Dunn’s post hoc test. For all remaining panels, statistical analysis was performed using one-way ANOVA followed by Tukey’s post hoc test. *p < 0.05, **p < 0.01. Statistical trends are indicated by adjusted p values.

**Figure 5.**
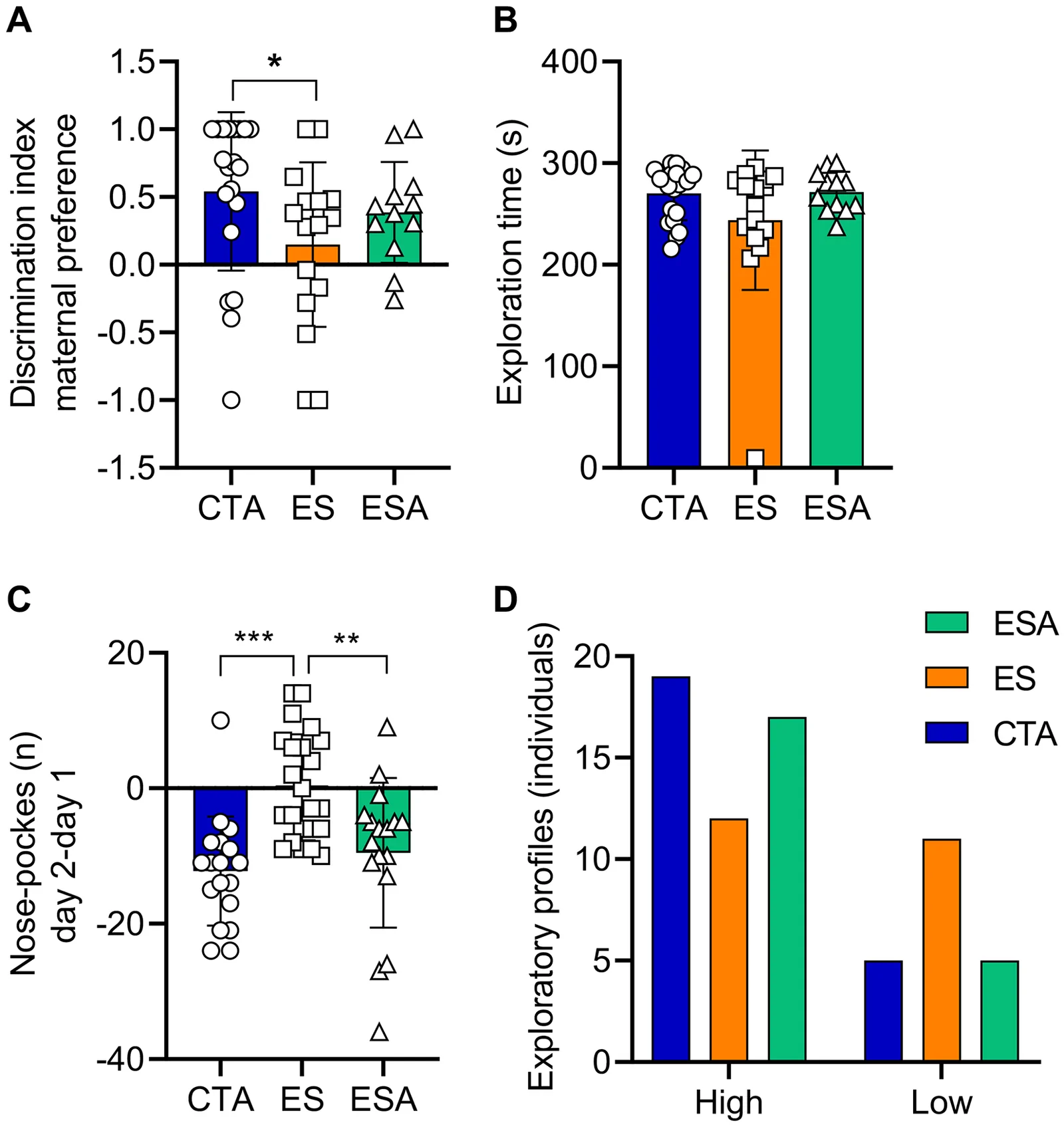
Evaluation of maternal bedding preference and spatial memory in offspring. Maternal bedding preference (**A** and **B**) and long-term spatial memory (**C**–**D**) were assessed in offspring. Discrimination index (**A**) and exploration time (**B**) are shown. Data are presented as mean ± SD (n = 10–12 per group). Statistical analysis was performed using the Kruskal–Wallis test (**A** and **B**), followed by the two-stage linear step-up procedure of Benjamini, Krieger, and Yekutieli (**A**) or Dunn’s post hoc test (**B**). The difference in the number of nose-pokes between day 2 and day 1 is shown for female offspring (**C**). Data are presented as mean ± SD (n = 12–20 per group). Statistical analysis was performed using one-way ANOVA followed by Tukey’s post hoc test (**C**). Exploratory profiles of offspring (**D**) are shown. Data are presented as the number of mice displaying either a high or low exploratory profile in each group (n = 11–12 per group). Statistical analysis was performed using the chi-square test.

**Figure 6.**
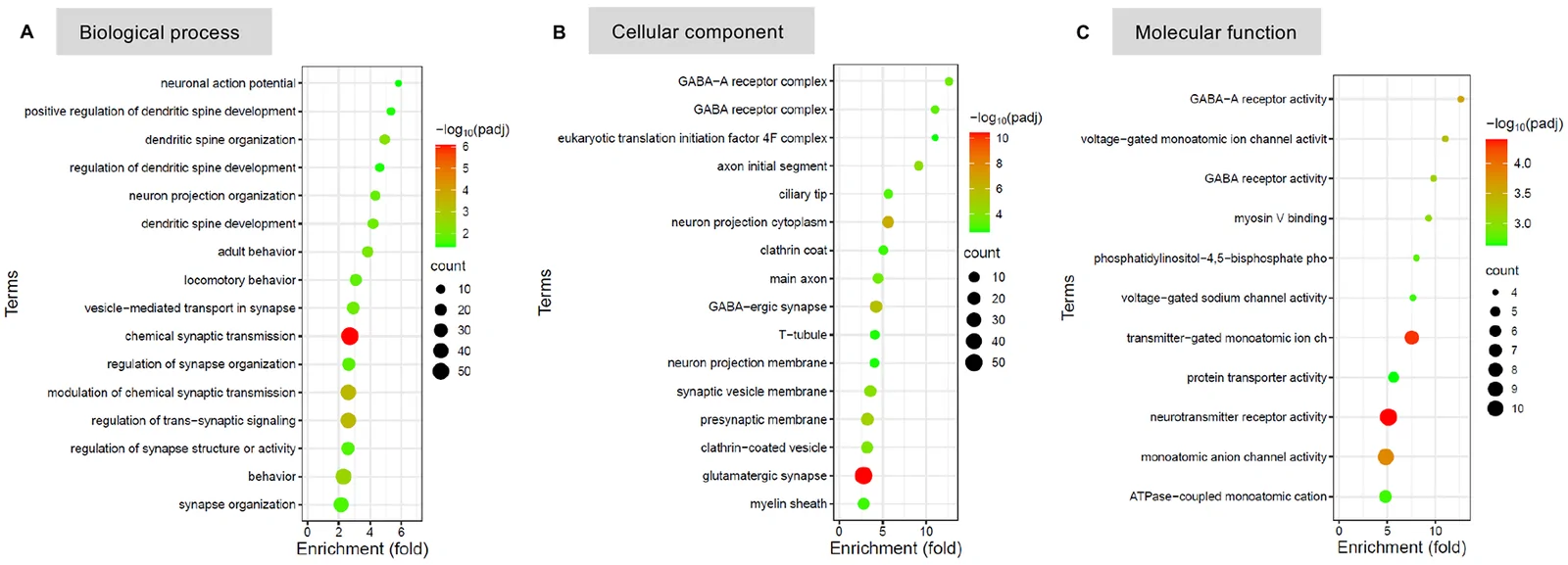
Gene Ontology (GO) enrichment analysis of differentially expressed genes identified by RNA sequencing. Gene Ontology (GO) enrichment analysis was performed on the differentially expressed genes identified by RNA-seq. Significantly enriched GO terms are shown for the three GO categories: Biological Process (A), Cellular Component (B), and Molecular Function (C). The x-axis indicates the enrichment fold for each GO term. Dot size represents the number of differentially expressed genes associated with each GO term (gene count), and dot color indicates the statistical significance of the enrichment (−log10 adjusted *p* value). GO terms are ranked according to their enrichment.

## Data Availability

The datasets used and/or analyzed during the current study are available from the corresponding author on reasonable request

## Abbreviations:

ABN: arched-back nursing
ACC: anterior cingulate cortex
BP: Biological Process
CC: Cellular Component
CTA: control group with social support
CT: control group
DEG: differentially expressed genes
EPM: elevated plus maze
ES: experimental stress group
ESA: experimental stress group with social support
FDR: false discovery rate
FST: forced swim test
GD1: gestational day 1
GSEA: gene set enrichment analysis
HPA: hypothalamic-pituitary-adrena
LG: licking and grooming
lncRNAs: long non-coding RNAs
mPFC: medial prefrontal cortex
MF: Molecular Function
NES: normalized enrichment score
NOR: novel object recognition
ORA: Overrepresentation analysis
PD: postnatal day
SI: social interaction
VTA: ventral tegmental area
